# Healthcare use by children in North West London 2015-2019 by deprivation and integrated care access

**DOI:** 10.1093/eurpub/ckac131.112

**Published:** 2022-10-25

**Authors:** F Neale, M Watson, D Hargreaves, S Arora

**Affiliations:** 1 Department of Primary Care and Public Health, Imperial College London, London, UK; 2 Department of Paediatrics, St Mary’s Hospital, London, UK

## Abstract

**Background:**

England has a health system offering universal coverage, but disparities in healthcare use are increasing. Between 2007-2017, children living in more deprived areas had higher rates of unplanned care (Emergency Department (ED) attendance and hospital admissions), whereas children from less deprived areas had higher rates of planned care (General Practitioner (GP) contact and outpatient appointments). More detailed research to find solutions for this divergent pattern is required.

**Aim:**

To assess the rates of GP contact, outpatient appointments, hospital admissions and ED attendance in North West London, by Index of Multiple Deprivation (IMD) decile of home postcode and access to an integrated care service providing linked care between multiple child health professionals ‘Connecting Care for Children (CC4C)', for children aged 0-18 years between 2015-2019.

**Methods:**

Retrospective analysis of a de-identified database of integrated care records for 495,357 children.

**Results:**

Children from the most deprived decile had higher rates of emergency admissions (0.070 per child per year vs. overall mean 0.040), elective admissions (0.076 vs. 0.032), ED attendances (0.754 vs. 0.358) and outpatient appointments (1.702 vs. 0.756) between 01.01.2015- 31.12.2019. Children from the least deprived decile had the second highest rates of outpatient appointments (0.911 vs. 0.756) and GP contact (8.192 vs. 5.390) between 01.01.2015- 31.12.2019. Children with access to the CC4C service, despite being from more deprived backgrounds, had lower rates of emergency admissions (0.028 vs. 0.037) compared to patients with access to usual care. P values <0.001 in all cases.

**Conclusions:**

Greater deprivation was linked to higher rates of emergency admissions, but this was partially mitigated by access to more integrated healthcare models. Children from the least deprived decile continued to have disproportionately higher use of planned care.

**Key messages:**

• Despite universal health coverage, children from more deprived areas continue to have disproportionately higher use of unplanned care.

• Further research is required to explore whether integrated care solutions can reduce the burden on unplanned health services and inequalities in access to care.

